# The correlation between alexithymia and communication skills among undergraduate pharmacy students

**DOI:** 10.1016/j.heliyon.2024.e41402

**Published:** 2024-12-20

**Authors:** Amjad H. Bazzari, Firas H. Bazzari

**Affiliations:** a Department of Basic Scientific Sciences, Faculty of Science, Applied Science Private University, Amman, 11931, Jordan; b Faculty of Pharmacy, Jerash University, Jerash, 26150, Jordan

**Keywords:** Social skills, Pharmacy practice, Feelings, Behavior, Education

## Abstract

Awareness of self-emotions and the emotions of others is a key factor in ensuring that health professionals communicate effectively with patients. This is of great importance for pharmacists, who, as primary healthcare providers, depend daily on effective communication to ensure the best therapeutic outcomes. Here, we aimed to evaluate the level of alexithymia, its correlation with communication skills and associated demographics in a university sample of undergraduate pharmacy students. The study utilized a printed questionnaire consisting of three parts: demographics, the Perth Alexithymia Questionnaire (PAQ), and the Health Professionals Communication Skills Scale (HP-CSS). A total of 212 students participated with a mean age of 21.59 ± 3.38 years, including males (n = 88, 41.5 %) and females (n = 124, 58.5 %). The results revealed moderate alexithymia levels and relatively satisfactory communication skills among the participants. Males had higher alexithymia scores and lower communication skills compared to females. Self-reported GPA values negatively correlated with alexithymia level but not with communication skills. The level of alexithymia negatively correlated with total communication skills, irrespective of gender, and the four dimensions of communication. In conclusion, alexithymia is negatively correlated with communication skills among undergraduate pharmacy students, highlighting the necessity of emotional awareness in patient communication and care.

## 1 Introduction

Pharmacists have key roles in primary healthcare and medication stewardship, with wide access to the public [1,2]. Their duties extend beyond dispensing medications to encompass a more integrative pharmaceutical care approach, which involves patient counseling and education, medication use review, collaboration with other health professionals, and overall promoting population health [3]. Pharmacists rely on effective communication skills daily to ensure safe and effective patient care [4]. As gatekeepers to medications, communication skills aid pharmacists in ensuring the administration of correct medications and proper dosing instructions, as well as identifying potential side effects and drug interactions that are all considered key elements for patient safety [5]. Furthermore, effective communication and strong interpersonal skills enable pharmacists to build trust with the patients, which, in turn, allows the patients to be more open in discussing some sensitive health-related information, promote their adherence to treatment plans, and increase their overall satisfaction with pharmaceutical care services [6]. Communication skills are also essential in a healthcare team setting to enhance collaboration with other healthcare providers to ensure better patient-centered care and effective therapeutic outcomes [7].

Emotional awareness is vital in communication, especially in pharmacy, where interactions with patients and other healthcare providers are frequent and sensitive [8]. Thereby, pharmacists must possess the ability to recognize, comprehend, and manage self and other's emotions to ease the interaction and ensure communication is clear, especially when dealing with individuals in distress who may be anxious, stressed, or generally upset [9]. Furthermore, emotional awareness can aid pharmacists in preventing misunderstandings by detecting signs related to confusion or discomfort and other indicators, such as voice tone and body language [10]. On the other hand, emotional awareness is critical in teamwork, as individuals can better deal with challenging work environments by reducing workplace tensions and promoting collaborative decision-making [11]. Therefore, incorporating training and dedicated courses to develop communication and affective skills in pharmacy curricula would aid in preparing students to meet the demands of modern pharmaceutical care services [12].

Alexithymia can be described as a personality trait that is indicated by difficulties in emotion recognition and regulation and an externally oriented thinking style with limited imaginal capacity, in other words, “no words for feelings” [13,14]. Studies assessing alexithymia levels among various populations have highlighted the relationship between increased alexithymia, reduced empathy, and communication and mental well-being problems [[15], [16], [17]]. Moreover, alexithymia was found to negatively impact the well-being of both the patients and healthcare providers; for instance, alexithymic patients were found to have higher physiological arousal and unhealthy compulsive behaviors [18]. In addition, alexithymia correlates with increased incidence of burnout, which is prevalent among pharmacists in Jordan [19], and depression as well as reduced social support among healthcare staff [20]. On the other hand, from an academic perspective, alexithymia is also linked to lower academic performance among college healthcare students, with significantly lower grade point average (GPA) levels among students with alexithymia [21]. Indeed, elevated alexithymia among college students negatively impacted self-efficacy, which is linked to motivation, achievement, and learning [22]. Therefore, assessing and addressing alexithymia is critical to improving academic performance, communication skills, and health outcomes.

The literature identified various factors associated with alexithymia, including, but not limited to, age, gender, socioeconomic status, and education [23]. Moreover, discrepancies in alexithymia levels were also linked to individuals’ country of residence, with several culture-related factors influencing living standards and social interaction patterns, among other things [24]. Furthermore, marital status and employment were also linked to alexithymia, as increased alexithymia rates were noticed among single and unemployed individuals [25].

So far, no study has been conducted to investigate whether alexithymia could impact communication skills among pharmacy students and pharmacists in Jordan. Thus, this study aims to evaluate the level of alexithymia and communication skills among pharmacy students, using validated scales, determine the existence of a correlation between them, and identify associated demographic factors. The findings could help answer whether alexithymia may negatively impact communication skills among undergraduate pharmacy students as future healthcare professionals and which aspects should be considered for future improvements.

## 2 Materials and methods

### 2.1 Study design

This cross-sectional study utilized a printed questionnaire (Appendix A), which was written in English and composed of three main parts: participant demographic factors, the Perth Alexithymia Questionnaire (PAQ), and the Health Professionals Communication Skills Scale (HP-CSS). The questionnaire was distributed among undergraduate pharmacy students enrolled in a Jordanian university.

### 2.2 Sampling and data collection

The eligibility criteria for participation were: adult age, undergoing field pharmacy training, and active enrolment in the pharmacy program, and 212 students participated. The researchers distributed the questionnaire during the students’ break hours at the university to provide all necessary information regarding the study and any clarifications required by the students.

### 2.3 Ethical considerations

Before handing out the questionnaire, each participant was provided with the necessary information regarding the goals and importance of the study. Participants were also informed that their privacy is protected, that the questionnaire will not include any identifying information questions, and that their answers will only be used for the purpose of scientific research. The participants were not offered or given any compensation for their participation and were aware that participation is voluntary and that they may withdraw at any point. All participants signed an informed consent to take part in the study.

The study's ethical approval was obtained from the Department of Pharmacy Council at Jerash University on the June 25, 2023 [approval number: mpc-004]. The study strictly adhered to the guidelines of the Declaration of Helsinki regarding studies involving human subjects [26]. The permission to use the HP-CSS [27] was obtained via email from Dr. César Leal Costa. Regarding PAQ [28], the authors Preece et al., 2018 have granted the freedom and permission to use it without needing contact to obtain permission, as highlighted in the PAQ: Scoring and Interpretation Manual [29].

### 2.4 Research instruments

#### 2.4.1 Demographics

The first section of the questionnaire involved participant demographics, that could potentially influence their alexithymia and communication skills levels. These included gender, age, marital status, working status, smoking status, nationality (Jordanian or international), permanent residence (Jordan or other), and self-reported GPA.

#### 2.4.2 Perth Alexithymia Questionnaire (PAQ)

The alexithymia level among the participants was assessed using the PAQ, which has an excellent internal consistency, assessed through two studies, with reported Cronbach's α values of 0.95 and 0.96 [28]. The PAQ is composed of 24 item questions scored using a 7-point Likert agreement scale with a 5-factored structure that assesses the total level of alexithymia and its main construct components: difficulty identifying one's own feelings (DIF); difficulty describing one's own feelings (DDF); and externally orientated thinking style (EOT), which involves lower attention focus to one's own feelings. Each question is scored from 1 to 7, and the total alexithymia score per participant is calculated as the sum of all question scores, leading to a potential total score range from 24 to 168, with higher scores indicating higher alexithymia levels. The scores of the PAQ subscales are calculated by summing the scores of their respective items: DIF of negative and positive feelings (N-DIF and P-DIF, respectively), DDF of negative and positive feelings (N-DDF and P-DDF, respectively), and general (negative and positive) EOT (G-EOT) subscale. Each subscale, except G-EOT, consists of 4 items (possible score range: 4–28), while G-EOT consists of 8 items (possible score range: 8–56). In addition to the total alexithymia score, five other composite scores are calculated: general DIF (G-DIF), general DDF (G-DDF), and the negative, positive, and general difficulty appraising feelings (N-DAF, P-DAF, and G-DAF, respectively). All composites, except total and G-DAF, have a potential score range from 8 to 56, while G-DAF ranges from 16 to 112.

#### 2.4.3 Health Professionals Communication Skills Scale (HP-CSS)

The communication skills by which health professionals relate to their patients were assessed using the HP-CSS, which exhibits high internal consistency, assessed through Cronbach's α, ranging from 0.65 to 0.78 for each of its four dimensions [27]. This scale consists of 18-item questions scored using a 6-point Likert frequency scale, with two items requiring inverse scoring. Each question is scored from 1 to 6, and the total communication skills score per participant is calculated as the sum of all question scores, leading to a potential total score ranging from 18 to 108, with higher scores meaning better communication skills. The scale exhibits a 4-factored structure that evaluates the four main dimensions of communication between the health provider and the patient. These include (1) informative communication (6 items, possible score range: 6–36), which reflects how the health professional obtains and provides information; (2) empathy (5 items, possible score range: 5–30), which reflects the capacity to comprehend patient feelings; (3) respect (3 items, possible score range: 3–18) shown by the health professional to the patient; and (4) social skills (4 items, possible score range: 4–24), such as being assertive.

### 2.5 Statistical analysis

JASP software (www.jasp-stats.org) was used for data analysis. The results of the study are reported as means or counts, along with their corresponding standard deviations (SD) or percentages (%), respectively. The association between gender and other participant demographics was tested using the Chi-square (χ2) test, except for continuous variables. The normality of distribution of these was tested using the Shapiro–Wilk test, which showed a significant result for age (P < 0.01). The *t*-test was used to compare GPA means, while the Mann-Whitney *U* test (MW test) was used to compare the age ranks of the participants. The rank-biserial correlation (rrb) and Cohen's d values were calculated to quantify the effect size for the differences in ranks and means, respectively. The internal reliabilities of the PAQ and HP-CSS were assessed using Cronbach's α value. The sums of participant answer scores, across all or select questions, were used to calculate their total and subscale scores, respectively. The differences and correlations of the scale scores, based on participant demographics, were tested using the MW test and Spearman's rank correlation test. The latter test was also performed to evaluate any correlations between the PAQ and HP-CSS scale measures and across their subscales. Lastly, all tests were two-tailed and performed using an α error of 5 %; thus, P < 0.05 indicates significance.

## 3 Results

### 3.1 Participant demographics

A total of 212 students participated, including males (n = 88, 41.5 %) and females (n = 124, 58.5 %). All responses were complete, and thus, all were included. The mean age of all participants was 21.59 ± 3.38 years and independent of gender (rrb = 0.04, P > 0.05). Most of the participants were single (88.21 %) in terms of marital status, and most were Jordanian by nationality (69.34 %) and reported Jordan as their location of permanent residence (73.11 %), with the rest being international students (30.66 %) or would be living abroad following graduation (26.89 %). The mean self-reported GPA was 79.31 ± 8.32 %, ranging from 60 % to 100 %, and was independent of gender (d = 0.25, P > 0.05) and other demographics. A significant difference based on gender, however, was observed for working status and smoking status (P < 0.01), with a higher ratio of working (44.32 %) and smoking (46.59 %) males compared to females (19.35 % and 13.71 %, respectively). All collected demographic factors are summarized in Table 1.

**Table 1:** Participant demographics.

Variable	Total	Male	Female	P value a
Age, mean (SD)	21.59 (3.38)	21.46 (2.94)	21.68 (3.66)	0.634
GPA, mean (SD)	79.31 (8.32)	78.11 (9.11)	80.17 (7.63)	0.075
Marital Status, n (%)				0.144
Single	187 (88.21 %)	81 (92.05 %)	106 (85.48 %)	
Married	25 (11.79 %)	7 (7.95 %)	18 (14.52 %)	
Smoking Status, n (%)				<0.001∗
Smoking	58 (27.36 %)	41 (46.59 %)	17 (13.71 %)	
Non-smoking	154 (72.64 %)	47 (53.41 %)	107 (86.29 %)	
Working Status, n (%)				<0.001∗
Working and studying	63 (29.72 %)	39 (44.32 %)	24 (19.35 %)	
Studying only	149 (70.28 %)	49 (55.68 %)	100 (80.65 %)	
Nationality, n (%)				0.224
Jordanian	147 (69.34 %)	57 (64.77 %)	90 (72.58 %)	
International	65 (30.66 %)	31 (35.23 %)	34 (27.42 %)	
Residence, n (%)				0.462
Jordan	155 (73.11 %)	62 (70.45 %)	93 (75 %)	
Other	57 (26.89 %)	26 (29.55 %)	31 (25 %)	

a Chi-square test was used for all comparisons across genders, except age (MW test) and self-reported GPA (independent *t*-test), ∗ Significant difference (P < 0.05).

### 3.2 Alexithymia level

The PAQ was used to assess the level of alexithymia, and exhibited an adequate level of internal reliability (Cronbach's α = 0.9). The total alexithymia scores were moderate (mean: 85.6 ± 25.86), ranging from 29 to 162, and were not normally distributed (SW statistic = 0.984, P < 0.05, skewness = 0.413, kurtosis = 0.257). The median alexithymia score was 83, and 27.4 % of the participants (n = 58) scored above 96, which is the midpoint of the possible scale range. The alexithymia subscale scores and their composites are provided in Table 2.

**Table 2:** Alexithymia subscale and composite scores.

Alexithymia subscales/composites	Items^a^	Mean	SD	Range^b^	Cronbach's α
Subscales					
Negative-Difficulty identifying feelings (N-DIF)	4	14.96	6.11	4–28	0.780
Positive-Difficulty identifying feelings (P-DIF)	4	12.94	5.63	4–28	0.752
Negative-Difficulty describing feelings (N-DDF)	4	15.93	5.87	4–28	0.729
Positive-Difficulty describing feelings (P-DDF)	4	13.68	5.68	4–28	0.739
General-Externally orientated thinking (G-EOT)	8	28.1	9.83	8–56	0.760
Composites					
General-Difficulty identifying feelings (G-DIF)	8	27.91	10.04	8–56	0.811
General-Difficulty describing feelings (G-DDF)	8	29.6	9.86	8–56	0.790
Negative-Difficulty appraising feelings (N-DAF)	8	30.89	10.28	8–56	0.806
Positive-Difficulty appraising feelings (P-DAF)	8	26.62	10.1	8–53	0.827
General-Difficulty appraising feelings (G-DAF)	16	57.51	18.62	16–106	0.884
Total Scale Score	24	85.6	25.86	29–162	0.900

a The number of items (each is scored from 1 to 7).

b Actual range of sample scores.

In regards to participant demographics, the total alexithymia scores differed by gender, with lower alexithymia levels among females (83.12 ± 25.88) compared to males (89.1 ± 25.57, rrb = 0.163, P < 0.05). In addition, alexithymia was found to negatively correlate with self-reported GPA values (ρ = −0.167, P < 0.05). A significant variation was also observed based on nationality and place of permanent residence. The total alexithymia score ranks were significantly lower for Jordanian participants (82.89 ± 24.54) and those whose permanent residence is Jordan (81.7 ± 24.32) compared to international students (91.74 ± 27.85, rrb = 0.195, P < 0.05) and participants who live abroad (96.21 ± 27.14, rrb = 0.32, P < 0.01), respectively. On the other hand, the total scores were independent of marital status, smoking status, and working status (P > 0.05).

### 3.3 Communication skills

The communication skills among the participants were quantified using the HP-CSS, which had a sufficient internal reliability (α = 0.82). The participants’ total scores ranged from 28 to 108 and were relatively satisfactory, with a mean of 83.64 ± 11.95. The median was 85, and 201 participants (94.8 %) had a total score above 63, which was the midpoint of the possible range of total scores (18–108). The total scores were not normally distributed (SW statistic = 0.95, P < 0.01, skewness = −0.96, kurtosis = 1.98). The HP-CSS total and dimension scores and relevant descriptives are provided in Table 3.

**Table 3:** Total and subscale communication skills scores.

Communication Skills Dimensions	Items^a^	Mean	SD	Range^b^	Cronbach's α
Informative Communication	6	28.21	4.44	11–36	0.558
Empathy	5	24.55	4.22	5–30	0.686
Respect	3	14.72	3.4	3–18	0.774
Social Skills	4	16.17	3.21	8–24	0.224
Total Score	18	83.64	11.95	28–108	0.816

a The number of items (each is scored from 1 to 6).

b Actual range of sample scores.

The total communication skills scores had significantly higher ranks for females (85.54 ± 11.49) compared to males (80.97 ± 12.14, rrb = 0.217, P < 0.01). It was the informative communication (28.95 ± 4.22 vs. 27.16 ± 4.56, rrb = 0.242, P < 0.01) and empathy dimensions (25.18 ± 4 vs. 23.66 ± 4.38, rrb = 0.218, P < 0.01) that differed by gender with higher scores for females, while respect and social skills were independent of gender (P > 0.05). Besides gender, the total communication scores did not differ by any other demographic factor.

### 3.4 Correlations

The two-tailed Spearman's rank correlation test was conducted to assess the correlations between the scale measures and across their subscale or composite scores. All five alexithymia subscales were positively and significantly (P < 0.01) correlated to each other to varying degrees with coefficients (rho, ρ) ranging from 0.32 (between G-EOT and N-DDF) to 0.69 (between N-DIF and P-DDF). Similarly, all four dimensions of communication skills were positively correlated to each other (ρ range: from 0.17 between social skills and respect, P < 0.05, to 0.65 between informative communication and respect dimensions, P < 0.01). The total alexithymia scores were negatively correlated to total communication skills (ρ = −0.315, P < 0.01), despite correcting for gender (ρ = −0.298, P < 0.01), and each of its four dimensions of communication (P < 0.05). Interestingly, except N-DDF (negative-difficulty describing feelings), all alexithymia subscale and composite scores as well were negatively correlated with communication skills total scores (P < 0.01). All correlation results between alexithymia and communication skills subscale and total scores are summarized in Table 4.

**Table 4:** Alexithymia and communication skills total and subscale score correlations.

Alexithymia Subscales		Dimensions of Communication Skills				
		Informative Communication	Empathy	Respect	Social Skills	Total Score
**N-DIF**	Rho^a^	−0.226	−0.047	−0.246	−0.127	−0.227
	p value	<0.001	0.493	<0.001	0.064	<0.001
**P-DIF**	Rho	−0.279	−0.307	−0.006	−0.142	−0.26
	p value	<0.001	<0.001	0.927	0.039	<0.001
**N-DDF**	Rho	−0.107	−0.136	0.139	−0.125	−0.103
	p value	0.121	0.048	0.043	0.069	0.135
**P-DDF**	Rho	−0.259	−0.065	−0.264	−0.089	−0.233
	p value	<0.001	0.349	<0.001	0.195	<0.001
**G-EOT**	Rho	−0.31	−0.255	−0.182	−0.202	−0.334
	p value	<0.001	<0.001	0.008	0.003	<0.001
**Total Score**	Rho	−0.307	−0.223	−0.152	−0.19	−0.315
	p value	<0.001	0.001	0.027	0.005	<0.001

a Spearman's rank correlation coefficient (rho, ρ).

## 4 Discussion

The current study is the first to evaluate the levels of alexithymia and communication skills and their relation among undergraduate pharmacy students in Jordan. The results showed moderate levels of alexithymia that differed by gender and correlated with GPA. The HP-CSS results obtained relatively satisfactory scores, which were higher for female compared to male participants. The alexithymia level displayed a negative correlation with total communication skills and its subscale scores.

The obtained alexithymia scores can be generally described as moderate and comparable to the results of other studies that adopted the PAQ among different populations. For instance, a total score of 84.37 was observed among Turkish nursing students [30] and 78.41 among Australian undergraduate psychology students [31], as well as 81.97 and 77.11 for Australian and United States adults, respectively [28,32]. Nonetheless, further improvements are warranted, considering the role of pharmacists as primary healthcare providers. There is a consistent link between alexithymia and difficulties in emotion regulation [33], and previous research showed that individual variations in differentiating negative emotions may play a significant role among multiple well-being indicators [34]. Several strategies were proposed to mitigate alexithymia; for example, self-control and mindfulness-based interventions have successfully reduced alexithymia among medical students [35]. Psycho-education has also been shown to reduce alexithymia and improve both emotional regulation and empathy among adolescents [36]. Implementing such strategies in pharmacy programs' curricula could benefit students immensely.

As for the HP-CSS, the obtained scores are relatively satisfactory, well above the midpoints of the possible score ranges, and comparable to previous literature that adopted the HP-CSS. For instance, a total mean of 83.98 was obtained among house job interns in Karachi [37], 91.97 among Chinese health professionals [38], and 87.1 among Spanish nursing students [39], which, all in all, indicates moderate to high performance. Nevertheless, there is still room for improvement in alexithymia mitigation and overall communication skill improvement. Multiple methodologies were proposed to enhance communication, social, and practice skills among pharmacy students, such as the implementation of simulated learning modules [40], incorporation of more clinical training courses/hours [41,42], and addition of role-plays and course(s) dedicated to communication skills and counseling [43], in addition to other strategies that can benefit in regards to social and communication skills competency among pharmacy students.

Concerning participant demographics, alexithymia scores negatively correlated with GPA. Recent studies examining the impact of alexithymia on academic performance have shown that alexithymic students are more likely to have negative feelings regarding their academic life and, in turn, influence their academic performance [44]. Moreover, the presence of other confounding variables among the students, such as childhood abuse and mental illness, increases the chances of having alexithymia [44]. This is in addition to the negative impact of alexithymia on self-efficacy among college students, which was suggested to be due to several factors; for example, the association between alexithymia and impaired interpersonal and communication skills that are both predictors of self-efficacy [22]. Therefore, it is well recommended to implement strategies to increase students' awareness regarding alexithymia and ways to seek help when needed, as well as early screening for alexithymia, which can all aid in reducing the potential negative consequences of alexithymia on students’ academic performance [22].

Regarding gender, it was associated with both alexithymia and communication skills. Male participants had higher alexithymia scores, consistent with previous research showing slight but consistent differences in alexithymia levels between genders [45]. Males also had lower overall communication skill levels than females, as previously reported for nurses using the HP-CSS scale [15]. The gender-based differences in healthcare provider-patient communication have been linked to various dissimilarities such as communication style, non-verbal communication, approach, exhibition of power, and interaction duration, length, and content [46,47]. These findings on the impact of gender and GPA may be expected to explain the negative correlation between alexithymia and communication skills observed in the current study; however, GPA was neither dependent on gender nor correlated to communication skills and the negative correlation between alexithymia and communication skills remained significant despite correcting for gender. This indicates that alexithymia indeed negatively impacts communication skills independently of gender or academic performance. This relationship is consistent with the literature [16,48], highlighting the necessity of alexithymia awareness and management in educational curricula.

The current study findings illustrate the negative impact of alexithymia on pharmacy students' communication skills, which has implications for education and practice. Accordingly, the results call for implementing targeted interventions within pharmacy education. Faculties of pharmacy should consider incorporating further training, courses, or workshops that cover and focus on emotional awareness and interpersonal skills development while continuously assessing their effectiveness in meeting the desired outcomes. Moreover, pharmacy programs should encourage the engagement of students in open dialogue activities, community outreach initiatives, role-plays, and team-based decision-making activities, as well as activities aiming to enhance cultural sensitivity that, all in all, contribute to the creation of supportive learning environments and develop students' skills and competencies. This is in addition to fostering students’ knowledge of trends in pharmaceutical care, such as patient-centered care, and the importance of interprofessional relationships that all require strong communication skills. In relation to pharmacy practice, the current findings recognize alexithymia as a barrier to effective communication, which may inspire further investigations regarding the prevalence and correlates of alexithymia among practicing pharmacists and other healthcare disciplines to assess the need for more comprehensive educational reforms as well as establish psychological support systems within universities and other sectors.

The article recognizes some limitations that need to be taken into consideration, and that may be addressed by future studies. Firstly, the results apply to pharmacy students in Jordan and may not accurately reflect other professions or populations. Secondly, the study was conducted on one university sample, and although the sample was collective in terms of demographics, variations among the students of other private or public universities in Jordan regarding alexithymia and communication skills may exist. Lastly, the study focused on undergraduate students of pharmacy and did not include those studying Doctor of Pharmacy (PharmD), which is a 6-year program of study also taught in Jordanian universities and involves hospital training and direct interaction with hospital inpatients. Future studies may consider the aforementioned in addition to the inclusion of more demographics and other related factors such as personality traits and interpersonal problems. Moreover, future studies should also focus on developing interventional strategies and investigating their effectiveness in raising emotional awareness and improving effective communication among students.

## 5 Conclusions

The level of alexithymia among undergraduate pharmacy students is moderate, dependent on gender and academic performance, as measured through GPA, and negatively correlated with total communication skills level as well as the various dimensions of health professional communication. The results highlight the importance of emotional awareness among pharmacy students regarding patient communication and, ultimately, patient care.

## CRediT authorship contribution statement

**Amjad H. Bazzari:** Writing – original draft, Software, Methodology, Investigation, Formal analysis, Data curation, Conceptualization. **Firas H. Bazzari:** Writing – review & editing, Methodology, Investigation, Conceptualization.

## Data availability statement

Data is available from the corresponding author upon reasonable request.

## Ethical approval statement

The Department of Pharmacy Council at Jerash University reviewed and approved this study, with the approval number [mpc-004], dated [June 25, 2023].

## Funding

No Funding was received.

## Declaration of competing interest

The authors declare that they have no known competing financial interests or personal relationships that could have appeared to influence the work reported in this paper.
