# Supplementary material for: The correlation between alexithymia and communication skills among undergraduate pharmacy students
Source: Heliyon. 2024 Dec 20;11(1):e41402. doi: 10.1016/j.heliyon.2024.e41402 (PMC11825255; doi:10.1016/j.heliyon.2024.e41402)
Supplement: Multimedia component 1 [file mmc1.pdf]

The Correlation Between Alexithymia and Communication Skills Among Undergraduate Pharmacy Students

QUESTIONNAIRE

Sec 1. Demographics:

1. Gender: _________________

2. Age: _________________

3. Grade point average (GPA): _________________

4. Marital Status:

☐ Single

☐ Married

5. Smoker:

☐ Smoking

☐ Non-smoking

6. Are you working in addition to studying:

☐ Working and studying

☐ Studying only

7. Nationality:

☐ Jordanian

☐ International

8. Place of Permanent Residence:

☐ Jordan

☐ Other

Sec 2. Perth Alexithymia Questionnaire (PAQ):

PAQ

Name: **Date:**

This questionnaire asks about how you perceive and experience your emotions. Please score the following statements according to **how much you agree or disagree that the statement is true of you**. Circle one answer for each statement.

Some questions mention *bad* or *unpleasant* emotions, this means emotions like sadness, anger, or fear. Some questions mention *good* or *pleasant* emotions, this means emotions like happiness, amusement, or excitement.

		Strongly disagree	----	----	Neither agree nor disagree	----	----	Strongly agree
1	When I'm feeling *bad* (feeling an unpleasant emotion), I can't find the right words to describe those feelings.	1	2	3	4	5	6	7
2	When I'm feeling *bad*, I can't tell whether I'm sad, angry, or scared.	1	2	3	4	5	6	7
3	I tend to ignore how I feel.	1	2	3	4	5	6	7
4	When I'm feeling *good* (feeling a pleasant emotion), I can't find the right words to describe those feelings.	1	2	3	4	5	6	7
5	When I'm feeling *good*, I can't tell whether I'm happy, excited, or amused.	1	2	3	4	5	6	7
6	I prefer to just let my feelings happen in the background, rather than focus on them.	1	2	3	4	5	6	7
7	When I'm feeling *bad*, I can't talk about those feelings in much depth or detail.	1	2	3	4	5	6	7
8	When I'm feeling *bad*, I can't make sense of those feelings.	1	2	3	4	5	6	7
9	I don't pay attention to my emotions.	1	2	3	4	5	6	7
10	When I'm feeling *good*, I can't talk about those feelings in much depth or detail.	1	2	3	4	5	6	7
11	When I'm feeling *good*, I can't make sense of those feelings.	1	2	3	4	5	6	7
12	Usually, I try to avoid thinking about what I'm feeling.	1	2	3	4	5	6	7

© **Preece et al. (2018):** Preece, D.; Becerra, R.; Robinson, K.; Dandy, J.; Allan, A. The psychometric assessment of alexithymia: Development and validation of the Perth Alexithymia Questionnaire. Pers. Individ. Differ. 2018; 132; 32–44. https://doi.org/10.1016/j.paid.2018.05.011

		Strongly disagree	----	----	Neither agree nor disagree	----	----	Strongly agree
13	When something *bad* happens, it's hard for me to put into words how I'm feeling.	1	2	3	4	5	6	7
14	When I'm feeling *bad*, I get confused about what emotion it is.	1	2	3	4	5	6	7
15	I prefer to focus on things I can actually see or touch, rather than my emotions.	1	2	3	4	5	6	7
16	When something *good* happens, it's hard for me to put into words how I'm feeling.	1	2	3	4	5	6	7
17	When I'm feeling *good*, I get confused about what emotion it is.	1	2	3	4	5	6	7
18	I don't try to be 'in touch' with my emotions.	1	2	3	4	5	6	7
19	When I'm feeling *bad*, if I try to describe how I'm feeling I don't know what to say.	1	2	3	4	5	6	7
20	When I'm feeling *bad*, I'm puzzled by those feelings.	1	2	3	4	5	6	7
21	It's not important for me to know what I'm feeling.	1	2	3	4	5	6	7
22	When I'm feeling *good*, if I try to describe how I'm feeling I don't know what to say.	1	2	3	4	5	6	7
23	When I'm feeling *good*, I'm puzzled by those feelings.	1	2	3	4	5	6	7
24	It's strange for me to think about my emotions.	1	2	3	4	5	6	7

© Preece et al. (2018)

© Preece et al. (2018): Preece, D.; Becerra, R.; Robinson, K.; Dandy, J.; Allan, A. The psychometric assessment of alexithymia: Development and validation of the Perth Alexithymia Questionnaire. Pers. Individ. Differ. 2018; 132; 32–44. https://doi.org/10.1016/j.paid.2018.05.011

Sec 3. Health Professionals Communication Skills Scale (HP-CSS):

	Almost never	Once in a while	Sometimes	Normally	Very often	Many times
I respect the right of patients to express themselves freelyb [*Respeto el derecho de los pacientes a expresarse libremente*].	☐	☐	☐	☐	☐	☐
I explore the emotions of my patients [*Exploro las emociones de mis pacientes*].	☐	☐	☐	☐	☐	☐
I respect the autonomy and freedom of patients [*Respeto la autonomía y libertad de los pacientes*].	☐	☐	☐	☐	☐	☐
When the patient speaks, I show interest through body gestures (nodding, eye contact, smiles, . . .) [*Cuando el paciente me habla muestro interés mediante gestos corporales (asintiendo con la cabeza, contacto ocular, sonrisas, . . .)*].	☐	☐	☐	☐	☐	☐
I provide information to patients (whenever my professional competency permits me) about what concerns them [*Proporciono información a los pacientes (siempre que mi competencia profesional me lo permita) sobre aquello que les preocupa*].	☐	☐	☐	☐	☐	☐
I listen to patients without prejudice, regardless of their physical appearance, mannerisms, form of expression, . . . [*Escucho a los pacientes sin prejuicios, independientemente de su aspecto físico, modales, forma de expresión, . . .*]	☐	☐	☐	☐	☐	☐
I express my opinions and desires clearly to patients [*Expreso claramente mis opiniones y deseos a los pacientes*].	☐	☐	☐	☐	☐	☐
When I give information, I use silence to allow the patient to assimilate what I am saying [*Cuando doy información, uso silencios para que el paciente asimile lo que le estoy diciendo*].	☐	☐	☐	☐	☐	☐

© **Leal-Costa et al. (2016):** Leal-Costa, C.; Tirado-González, S.; Rodríguez-Marín, J.; Vander-Hofstadt-Román, C.J. Psychometric properties of the health professionals communication skills scale (HP-CSS). Int. J. Clin. Health Psychol. 2016; 16; 76–86. https://doi.org/10.1016/j.ijchp.2015.04.001

	Almost never	Once in a while	Sometimes	Normally	Very often	Many times
When I give information to patients, I do so in understandable terms [*Cuando doy información a los pacientes lo hago en términos comprensibles*].	☐	☐	☐	☐	☐	☐
When a patient does something that does not seem right, I express my disagreement or discomfort [*Cuando un paciente hace algo que no me parece bien le expreso mi desacuerdo o molestia*].	☐	☐	☐	☐	☐	☐
I dedicate time to listen and try to understand the needs of patients [*Dedico tiempo para escuchar y tratar de comprender las necesidades de los pacientes*].	☐	☐	☐	☐	☐	☐
I try to understand the feelings of my patient [*Trato de comprender los sentimientos de mi paciente*].	☐	☐	☐	☐	☐	☐
When I interact with patients, I express my opinions clearly and firmly [*Cuando me relaciono con los pacientes expreso mis comentarios de una manera clara y firme*].	☐	☐	☐	☐	☐	☐
I believe that the patient is entitled to receive health information [*Considero que el paciente tiene derecho a recibir información sanitaria*].	☐	☐	☐	☐	☐	☐
I feel that I respect the needs of patients [*Siento que respeto las necesidades de los pacientes*].	☐	☐	☐	☐	☐	☐
I find it difficult to make requests of patients [*Me resulta difícil realizar peticiones a los pacientes*].	☐	☐	☐	☐	☐	☐
I make sure that patients have comprehended the information provided [*Me aseguro que los pacientes han comprendido la información proporcionada*].	☐	☐	☐	☐	☐	☐
I find it difficult to ask for information from patients [*Me resulta difícil pedir información a los pacientes*].	☐	☐	☐	☐	☐	☐

© **Leal-Costa et al. (2016):** Leal-Costa, C.; Tirado-González, S.; Rodríguez-Marín, J.; Vander-Hofstadt-Román, C.J. Psychometric properties of the health professionals communication skills scale (HP-CSS). Int. J. Clin. Health Psychol. 2016; 16; 76–86. https://doi.org/10.1016/j.ijchp.2015.04.001
